# A Spiking Neural Network Based on Retinal Ganglion Cells for Automatic Burn Image Segmentation

**DOI:** 10.3390/e24111526

**Published:** 2022-10-25

**Authors:** Jiakai Liang, Ruixue Li, Chao Wang, Rulin Zhang, Keqiang Yue, Wenjun Li, Yilin Li

**Affiliations:** Zhejiang Integrated Circuits and Intelligent Hardware Collaborative Innovation Center, Hangzhou Dianzi University, Hangzhou 310018, China

**Keywords:** burn image, image segmentation, spiking neural network, retinal ganglion cells

## Abstract

Burn is a common traumatic disease. After severe burn injury, the human body will increase catabolism, and burn wounds lead to a large amount of body fluid loss, with a high mortality rate. Therefore, in the early treatment for burn patients, it is essential to calculate the patient’s water requirement based on the percentage of the burn wound area in the total body surface area (TBSA%). However, burn wounds are so complex that there is observer variability by the clinicians, making it challenging to locate the burn wounds accurately. Therefore, an objective, accurate location method of burn wounds is very necessary and meaningful. Convolutional neural networks (CNNs) provide feasible means for this requirement. However, although the CNNs continue to improve the accuracy in the semantic segmentation task, they are often limited by the computing resources of edge hardware. For this purpose, a lightweight burn wounds segmentation model is required. In our work, we constructed a burn image dataset and proposed a U-type spiking neural networks (SNNs) based on retinal ganglion cells (RGC) for segmenting burn and non-burn areas. Moreover, a module with cross-layer skip concatenation structure was introduced. Experimental results showed that the pixel accuracy of the proposed reached 92.89%, and our network parameter only needed 16.6 Mbytes. The results showed our model achieved remarkable accuracy while achieving edge hardware affinity.

## 1. Introduction

Burns generally refer to structural damage to the skin tissue caused by heat, including hydrothermal fluids, hot gases, and flames. Minor burns may cause subcutaneous or mucosal tissue damage. Severe burns may enhance the permeability of capillaries on the wound surface, causing excessive body fluid leakage, massive loss of electrolytes, and being accompanied by shock and multiple organ failure, which are leading causes of death from burns [1]. Therefore, early adequate resuscitation fluid supplementation ensures the life safety of burn patients, which is very important for preventing and treating burn shock and other heat injury complications [2]. Currently, the severity of burns and calculation of required resuscitation fluid is largely determined by estimates of the percentage of body surface burn area (TBSA%) [3]. In clinical treatment, within 24 h of burns, the resuscitation fluid required for each hour of post-shock resuscitation is most widely described by the Parkland formula [4]. In a nutshell, resuscitation fluid usage is calculated based on TBSA% and the patient’s weight (TBSA% × weight [kg] × 4 [mL]/24[H]). Therefore, accurate assessment of TBSA% has become a key task for the early treatment of burns.

In general, the most accurate way to calculate TBSA% is by drawing the burn area based on the Lund and Browder chart [5]. In addition, Rule of Nine’s burn area charts [6] also is widely used to estimate TBSA% in clinical practice. However, studies had shown that burn experts and burn surgeons used Lund and Browder chart to estimate TBSA with significantly higher reliability than burn nurses and other temporary estimators. Similarly, the reliability of the nine-rule chart had more significant differences among different evaluators [7,8]. In addition, to improve speed, clinicians usually rely on palm area to estimate TBSA%, where a palm-size burn area is about 1% in general. However, the accuracy of this method is different for the same patient due to the different size of the assessor’s palm [9]. In recent years, some burn area diagnostic technology and equipment have been developed, such as laser doppler imaging [10], harmonic ultrasound imaging [11], and high-resolution infrared thermal imaging techniques [12]. These techniques help general practitioners or nurses to calculate TBSA% and reduce the diagnostic gap between observers. However, all equipment applied and the mentioned methods above were tricky to be widely used in general hospitals due to strict operating conditions and high costs. Therefore, the development of a lightweight and accurate burn area segmentation method for portable equipment has also become one of the challenges.

In the early research of burn area segmentation, Zhao et al. [13] proposed a structure composed of preprocessing algorithm by analyzing the multipath spectra in visible and near-infrared areas. Combined with principal component analysis (PCA) and a hybrid neural network, local thickness and total thickness (burn depth grade) classification accuracy was 87.5%. Wantanajittikul et al. [14] used CR transform, Luv transforms, and fuzzy c-means clustering to separate burn wounds and healthy skin areas and used mathematical morphology to improve the separation accuracy. They used h-transformation and texture analysis to extract feature vectors as inputs of a support vector machine (SVM) to identify the degree of burn. Their experimental results have shown that the classification accuracy was 75.33%. Acha et al. [15] proposed a space with the multidimensional scaling (MDS) axes to determine the physical characteristics that physicians employ to diagnose a burn depth. K Nearest Neighbor (KNN) was used to classify the burn depth through these characteristics, and the success rate was 83.8%. However, these traditional methods often fail to achieve satisfactory accuracy.

In recent years, convolutional neural networks (CNNs) have made significant progress in machine vision and the field of medical image analysis [16]. More researchers have been trying to apply CNNs to burn image segmentation. Chauhan et al. [17] customized a specific burn severity estimation (BSE) model based on different body parts to solve the interference of skin characteristics with an accuracy of 84.85%. At the same time, Liu et al. [18] proposed an end-to-end framework for burn depth diagnosis based on a high-resolution network (HRNetV2) with C1 (one convolution module). Finally, in the segmentation of burned and unburned areas, intersection-over-union, pixel accuracy, and dice coefficient indexes reached the results of 0.8467, 0.9459, and 0.9170, respectively.

However, although CNNs are continuously deepened to improve their performance, it still has some limitations. First, the ever-deepening CNNs not only improves the accuracy but also challenges the computing power of the computer. This means that CNNs become difficult to obtain good terminal-friendly performance while ensuring the accuracy of the algorithm [19]. Second, CNNs usually needs more feature dimensions to process images containing complex information. Burn images usually contain complex external features of burn wounds [20], which means that using CNNs to segment burn areas requires deeper structures and larger parameters. Moreover, due to the lack of research on burns, there were no public datasets about burn images on the Internet [21]. CNNs will suffer from overfitting and high square error gradient when facing high dimensional, low sample size (HDLSS) data [22]. This not only runs counter to our demand for lightweight methods but also fails to achieve satisfactory results. Therefore, finding a lightweight and accurate burn region segmentation method suitable for complex burn images has become the focus problem of burn area segmentation in burn treatment.

In recent years, spiking neural networks (SNNs) have become a popular third-generation artificial neural network due to their brain-inspired computing structure [23] and hardware-friendly characteristics. In SNNs, leaky integrate-and-fire models (LIF) [24] and spike-timing-dependent plasticity (STDP) [25] were widely used to simulating neural dynamics to ensure the proper performance of the network, which was more reasonable in biology than CNNs. Similarly, based on these models, neural morphological hardware circuits suitable for parallel and distributed computing are also proposed to meet the requirements of computing performance and low power consumption [26]. SNNs uses sparse and asynchronous methods to process binary spike signals, which makes SNNs usually consume less energy than CNNs to implement edge computing in hardware [27]. Moreover, SNNs had shown exemplary performance in many computational vision tasks. For example, Qian et al. [28] proposed a spiking neural network with unsupervised STDP, with an efficient temporal encoding and winner-take-all (WTA) mechanism. This SNN classed malignant melanoma and benign melanocytic nevi with an average accuracy of 83.8%. Rueckauer et al. [29] proposed an efficient time coding scheme, which took the simulated activation of neurons in an analog neural network (ANN) as SNN time-to-first-spike (TTFs). They demonstrated that the computation on MNIST was 7–10 times less than the original simulation model, and the accuracy loss was less than 1%. Lee et al. [30] proposed an SNN error back-propagation mechanism. This mechanism similar to traditional deep neural networks directly acts on membrane potential and spikes and achieves an accuracy of 98.77% on MNIST. However, at present, the application of SNNs in complex scenes is still rare, and it has not been reported in the burn image segmentation that this paper focuses on.

Therefore, this paper constructs a burn image dataset, and designs a SNN model based on RGC to realize burn image segmentation. Our contributions of this paper were as follows:

(1) Organizing burn image datasets.
(2) A burn area segmentation method with few parameters was proposed.
(3) The retinal ganglion cells, which neuron dynamics are more in line with the characteristics of the brain, were introduced into our SNN model.
(4) An improved input coding method and the cross-layer skip concatenation structure was introduced to our SNN model.

## 2. Methods

**Figure 1:**
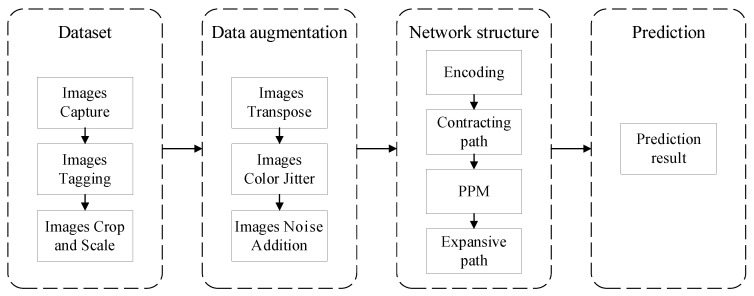
The framework of the burn area segmentation system in this study.

Figure 1 shows the framework of our burn area segmentation system. We first constructed the burn image dataset. Data augmentation was then performed on the burn image dataset. After that, the augmented images were fed into our SNN based on retinal ganglion cells (RGC), called as RGC-SNN, proposed in this paper for training. Finally, prediction results were made on the test dataset. The detailed contents of these methods are described in the next subsections. 

### 2.1. Dataset

**Figure 2:**
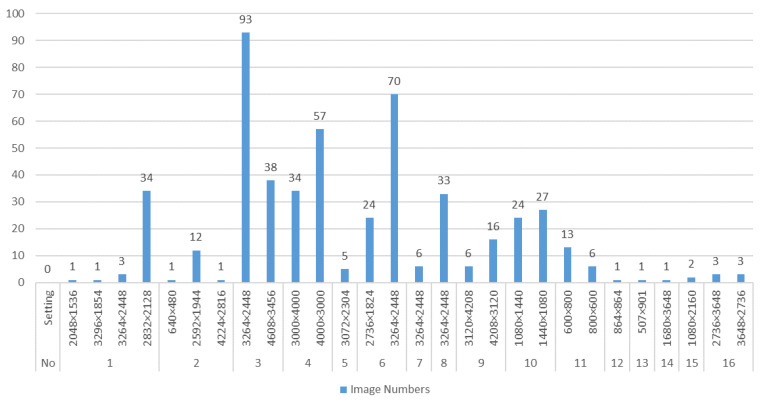
The number of burn images collected by equipment NO.1 to NO.16 with different pixel parameter settings. Among them, NO.1 to NO.9 were camera devices, and NO.10 to NO.16 were mobile devices.

Our study was supported by the Hangzhou (China) Jianggan District People’s Hospital. With the patients’ permission, we selected several different models of cameras or smartphones to take photographs of burn areas to form 481 patients at various angles and light intensity, increasing the diversity of the samples. These subjects included 268 males and 213 females, ranging from 5 to 65 years old. They suffered varying degrees of burns covering several parts of their body including head, torso, arms, and legs. The diversity of their skin conditions caused by differences in gender and age contributed to the diversity of dataset. Finally, 516 unprocessed images of burn patients were captured. Due to different camera models and pixel parameter settings, the image size is different. The details of the number of images of burn patients collected by different models of equipment and the setting of pixel parameters are shown in Figure 2. Most of the pictures had resolutions of 3264 × 2448 and 3000 × 4000, and were taken by Nikon camera and Sony cameras. The resolution of the pictures taken by the mobile phone was relatively small, with a total of 81 images.

**Figure 3:**
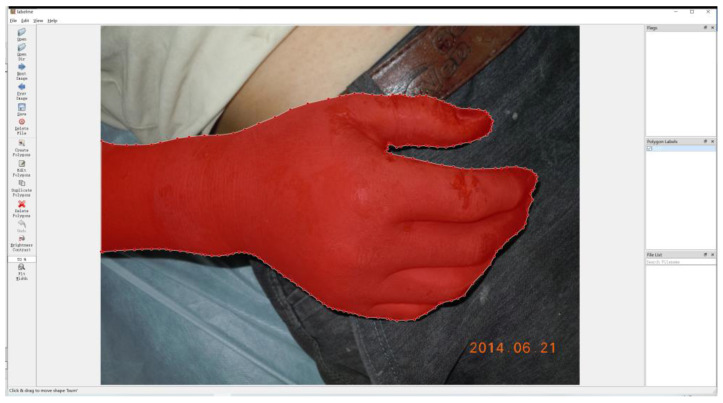
The labeled burn images with the LabelMe software. The inside of the red polygon was marked as burn, and the outside of the red polygon was marked as non-burn.

In these images, the burn areas were labelled by two specialists with the help of LabelMe [31]. As shown in Figure 3, the area inside the red dot line was burn part, labelled as “0” in corresponding pixels; and other areas including unburned skin, clothing, and background environment were marked as “1”. Furthermore, in order to improve the computational efficiency and increase the number of training samples, the annotated burn image was cropped. During the cropping process, the number of labels “0” and “1” in a single image was kept as close as possible, and there was no overlapping area in the cropped image. Annotated burn image from an average resolution of 3063 × 2479 was cropped into a smaller image of 1331 × 1677.

**Figure 4:**
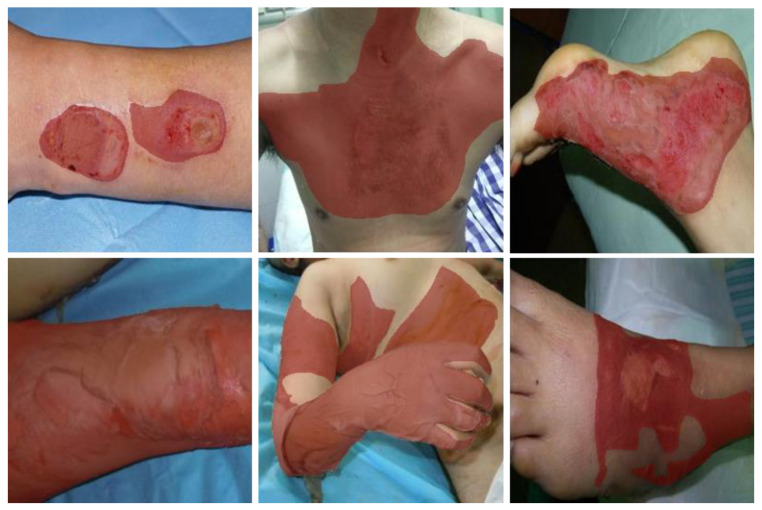
Example of a partial burn image dataset. The red mask indicates the burned area.

Finally, we obtained 1200 burn images with annotations, which were divided into a training dataset including 1000 images and a test dataset for the left. Lastly, they were uniformly resized to 256 × 256 to facilitate subsequent calculation. Figure 4 shows some examples of the burn image dataset.

### 2.2. Data Augmentation

**Figure 5:**
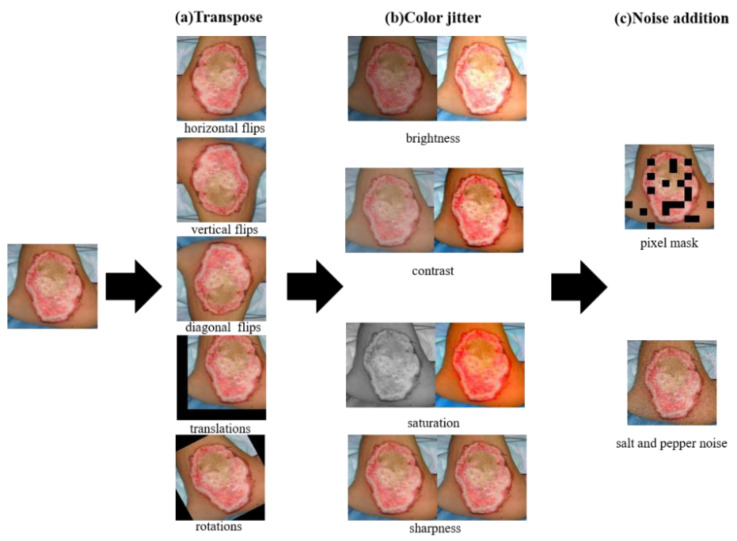
Data augmentation.

Data augmentation was used to improve the generalization ability of the network model and prevent overfitting [32]. In this paper, we applied three augmentation methods: transposition, color jitter, and noise addition to enrich our burn image dataset. Figure 5 shows an example to illustrate the three image enhancement methods.

The transposition methods included random flips, translations, and rotations. The translations range was from 100 pixels to −100 pixels in the horizontal and vertical directions. The rotations angle was between 90 degrees and −90 degrees. After transposition methods, the number of images increased five times, as shown in Figure 5a.

Furthermore, in order to adapt the model to more working scenes, four color jitter methods were used to change the brightness, contrast, saturation, and sharpness of the burn image respectively. We set an enhancement factor for four color jitters, where enhancement factor 1 represented the original image. Brightness was used to control the brightness of an image, where an enhancement factor of 0 represented a black image. The contrast was used to control the contrast of the image, where the enhancement factor of 0 represented the saturation of the gray image. Saturation was used to adjust the color balance of the image, where an enhancement factor of 0 represented a black-and-white image. Sharpness was used to adjust the sharpness of the image, where the enhancement factor less than 1 meant blurring the image, and the factor greater than 1 meant sharpening the image. During training, the brightness and contrast enhancement factors of each batch were random between 0.5 and 1.5, and the saturation and sharpness enhancement factors were random between 0 and 2. After color jitter methods, the number of images increased four times. As shown in Figure 5b.

Finally, salt and pepper noise and pixel mask were added to the image to eliminate the influence of noise caused by acquisition devices with different pixel settings on the prediction results. In total, we obtained a burn image dataset including 20,000 images, which was 20 times the size of the original dataset. 

### 2.3. Network Structure

**Figure 6:**
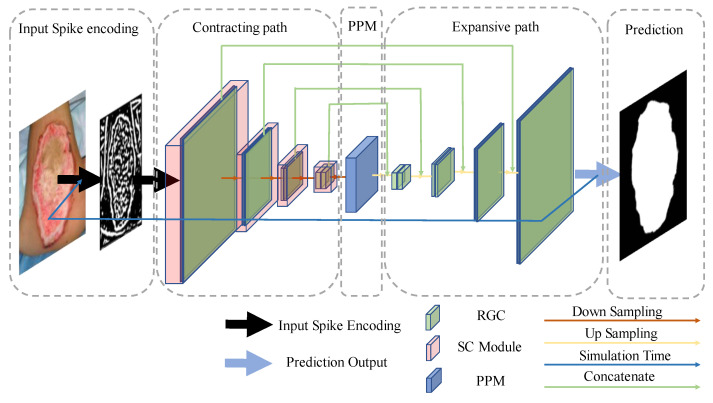
The structure of our RGC-SNN model in this study.

In semantic segmentation tasks, the U-type structure [33] was widely used. In this paper, we designed a U-type RGC-SNN for the burn image segmentation, which detailed architecture was shown in Figure 6. The network consisted of four main parts: input spike encoding, contracting path, pyramid pooling module (PPM), and expansive path.

#### 2.3.1. Retinal Ganglion Cell Neuron

RGC model was summarized by Sernagor et al. [34] RGC process and convey information from the retina to visual centers in the brain. In our work, we introduced RGC as a neuron to enrich the dynamics of neurons in the SNN.

Based on the research of Sernagor et al., the dynamic of the membrane potential of RGCs obeyed Kirchhoff’s Law as follows:

(1) $$C\frac{dU}{dt}=-U+\sum_{i}I_{i}+I\left(t\right)$$

(2) $$\sum_{i}I_{i}=g_{\mathrm{Na}}m^{3}h\left(U-E_{Na}\right)+g_{K}n^{4}\left(U-E_{K}\right)+g_{L}\left(U-E_{L}\right)$$

where U was the membrane potential, dt was the time difference. $\sum_{i}I_{i}$ was the ion channel current of integrating sodium current, potassium current, and leaky current, as described in Equation (2). Here, $E_{Na,K,L}$ were the equilibrium potential of sodium ion, potassium ion, and leaky ion, respectively. $g_{Na,K,L}$ were the ion channel conductivity of sodium ion, potassium ion, and leaky ion, respectively. Gating variables m, n, h represented the probability of sodium ion channels and potassium ion channels open. I(t) was the external input. 

In our work, the spike discharge threshold was set for the membrane potential to judge whether the membrane potential exceeds the threshold. In this paper, the threshold was set as 0.5. If the membrane potential exceeds the threshold, RGC generated a spike discharge, and the membrane potential was reset to the value of $U-V_{th}$. The change of the membrane potential is described as follows:

(3) $$U=\left\{ \begin{array}{l} U-V_{th}\;and\;Spike=1,\;if\;U>V_{th}\\ U,\;if\;U<V_{th} \end{array}\right.$$

In the calculation process, the Equation (3) was converted into the iterative Equation (4):

(4) $$U^{T,N}=U^{T-1,N}+\sum_{j}\omega o{\left(j\right)}^{T,N-1}-{\sum_{k}I_{k}}^{T,N-1}-(1-spike^{T-1,N})V_{th}$$

where T was simulation time step and N was the index of RGC-SNN layers.

#### 2.3.2. Input Spike Encoding

The role of the input spike coding in the spiking neural network was to build a bridge between the real value of the input image and SNNs. So far, researchers have proposed a variety of coding methods. For example, in order to make full use of time coding, the time-to-first-spike (TTFs) coding has been applied to SNN [29,35]. Nitin Rathi et al. proposed that the input layer directly processed the analog pixel value of the image without converting it into a spike sequence [36]. Qi Xu et al. [37] proposed a CNN-SNN (CSNN) model which used perceptron to encode real values into spikes. It had excellent learning ability, coding mechanism, robustness to noise stimulation, and performance, which brought more biological realism to modern image classification models. A pre-trained convolutional layer and a pooling layer were composed to form a perceptron, where the pooling layer generated a set of activation levels analog values. Finally, these analog values were converted into coefficient discharge signals by the threshold, time coding, and other coding methods.

**Figure 7:**
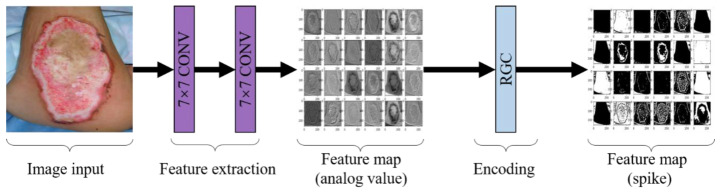
Convolutional spike coding structure and process.

In our study, we also used convolutional layers to constitute a perceptron, but it was regarded as a part of our RGC-SNN to be trained instead of a fixed module in CSNN. Moreover, the temporal encoding was replaced by RGC. Our perceptron contains two parts: a double convolution module and some RGC neurons. Each pixel value in the burn image was converted into a real value by perceptron to simulate the external current of neurons in input layer and was encoded into spikes by RGC eventually. Among the double convolution modules, two convolutional layers with a kernel size of 7 × 7 were set to extract large-scale features from burn images. Furthermore, the channel number of the output feature maps both were set to 24. The detailed architecture of the perceptron is shown in Figure 7.

**Figure 8:**
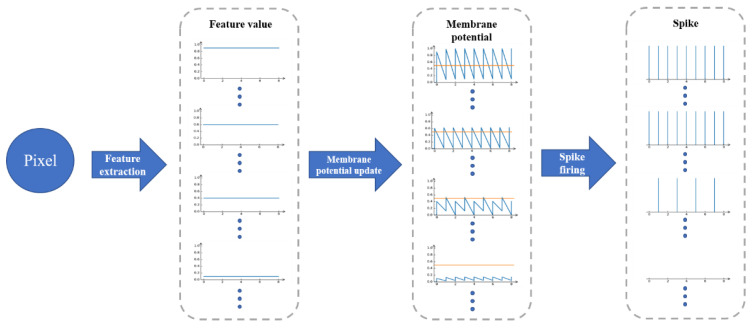
The coding process shown how a pixel value to fire spike.

Here, to show the encoding process more clearly, we used a pixel value as an example. After feeding the pixel value into the encoder, the feature extraction module generates 24 feature value to represent the activation intensity of the corresponding spikes. We took some of 24 feature value and draw their coding process, as shown in Figure 8. These feature value simulated the activation intensity analog values of the external input currents I(t) of RGC neurons, which were input into the RGC layer to update membrane potential. The update law of membrane potential follows the Equation (1).

After the membrane potential was updated, spike firing mechanism was implemented. The fire of the spike was judged by Equation (1) according to the membrane potential. In Figure 8, the yellow line in the membrane potential column represents the value of firing threshold. 

In this paper, we set eight simulation time steps for RGC to simulate the updating-firing mechanism. After that, the pixel value was converted to spike train.

Finally, each 256 × 256 burn image was encoded into 24 × 256 × 256 feature maps, where each pixel value was 0 or 1, as the input of the subsequent structure of RGC-SNN. 

#### 2.3.3. Contracting Path

**Figure 9:**
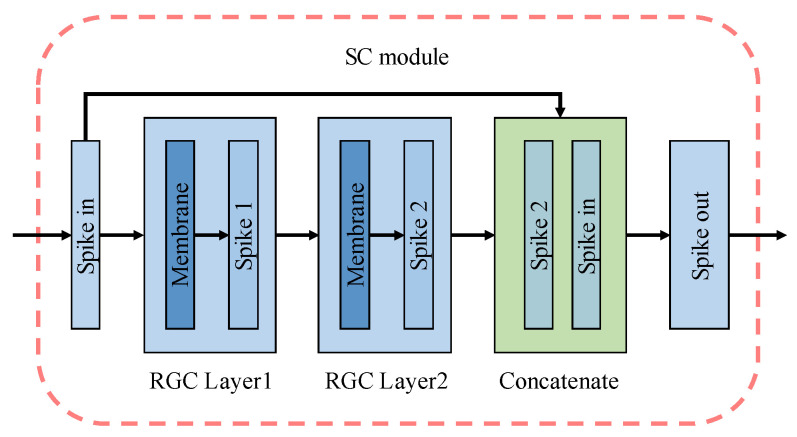
The details of skip concatenation module.

The primary function of the contracting path was to extract the critical features in the encoded spike. Considering that SNNs may had the problems of feature disappearance or network degradation in the process of spikes transmission, a module with cross-layer skip concatenation (SC) structure was introduced to simulate remote connections in the nervous system, as shown in Figure 9, into our RGC-SNN model to reuse shallow features to prevent overfitting or vanishing gradient problems caused by deepening of SNNs model. Our SC module was fitted by two RGC firing neuron processing layers (blue layer) and a concatenate layer (green layer). We consider the structure defined as:

$$Spike_{out}=Concatenate(RGC_{layer2}\left(RGC_{layer1}\left(Spike_{in}\right)\right)\;,\;Spike_{in})$$

here $Spike_{in}$ and $Spike_{out}$ were the input and output of the SC module. SC module concatenates two feature maps in the channel dimension. The concatenate layer played the role of cross layer connection to realize the function of remedying the disappearance of features. 

**Figure 10:**
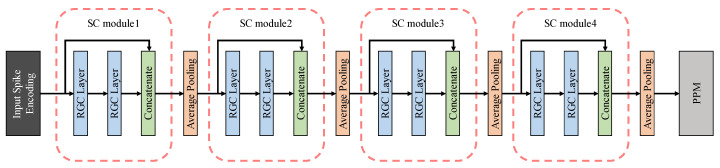
Contracting path and SC modules.

In this paper, the contraction path was made up of ten layers, shown in Figure 10, including input layer and output layer, and SC modules. In this paper, the contraction path contains four SC modules, and each module was followed by an average pooling layer with a step size of 2 × 2 as the transition structure. The encoded visual information (24 × 256 × 256, which means channels × height × width) was characterized as feature maps (384 × 16 × 16) by the contraction path and input them into the pyramid pooling module. we introduce the pyramid pooling module in detail in Section 3.3. 

#### 2.3.4. Pyramid Pooling Module

**Figure 11:**
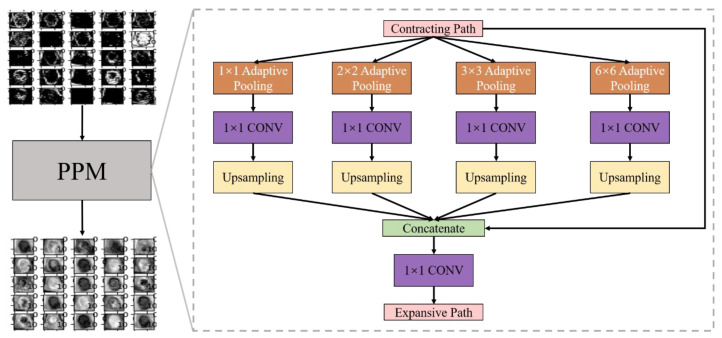
Partial channel feature maps display and PPM specific structure.

In our work, in order to better process the global and local information in the burn image dataset, after the contraction path, the image feature maps were sent to the pyramid pool module (PPM) layer. PPM was a part of the pyramid scene parsing network proposed by Zhao et al. [38]. Their research showed that ppm effectively improved receptive field and global information utilization through pool operation at different scales. Figure 11 shows the PPM used in this paper, which consists of four branches: four adaptive pooling layers (orange-yellow layer) with target sizes of 1 × 1, 2 × 2, 3 × 3, and 6 × 6, respectively. After each adaptive pooling layer, a convolution layer (purple layer) with a kernel size of 1 × 1. PPM performed pool and convolution operations on the feature maps. Then these feature maps size are restored by bilinear interpolation upsampling layers (yellow layer). Finally, the feature maps of these four branches were connected by the concatenate layer with the input feature maps of ppm. The feature maps (768 × 16 × 16) after concatenation layer was compressed by the convolution layer with the convolution kernel of 1 × 1 to obtain the final composite feature maps (384 × 16 × 16). Those composite feature maps were sent to the expansive path by us.

#### 2.3.5. Expansive Path and Predication

**Figure 12:**
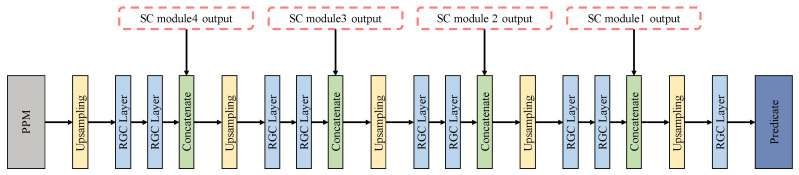
Expansive path and predication.

In our work, the expansive path was connected after PPM. The primary function of the expansive path was to restore the predicted characteristic image to the size of the input image and output the predicted value. There were 20 layers in total, which are shown in Figure 12, including concatenate layers (green layer) and the final prediction layers (dark blue layer). The SC module’s output (in the dotted box in Figure 12) in the contracting path were connected by the concatenate layers. Concatenate layers fuse shallow location features with deep semantic information, and alleviate the problem of gradient disappearance to a certain extent. The final prediction layer outputs the prediction results. Feature maps (2 × 256 × 256) restored by the expansion path were entered into the final prediction layer. The model predicted the output feature maps on the 20th layer of the expansive path. The category with more discharge times in simulation time was counted as the prediction category.

### 2.4. Learning Strategy and Loss Function

This paper adopted the back-propagation learning strategy to complete the training of the SNN network. We know that the derivative of a spike was zero-valued everywhere except at excitation point, which causes the gradient in backpropagation to vanish or explode. To solve this problem, we introduced the gradient approximation Equation (5) to approximate the spike gradient function [39].

(5) $$h_{1}(U)=\frac{1}{a_{1}}sign(|U-V_{th}|<\frac{a_{1}}{2})$$

where we set $a_{1}$ to 1, to ensure that the integral of the gradient approximation function was 1. 

In this paper, cross-entropy (CE) loss was used for the training networks. The CE loss function was

(6) $${\mathrm{Loss}(y}_{\mathrm{pred}}{,y}_{\mathrm{true}})=-\sum_{class}y_{\mathrm{true}}\log(y_{\mathrm{pred}})$$

where $y_{\mathrm{pred}}$ was the output prediction value of RGC-SNN and $y_{\mathrm{true}}$ was the label value of dataset.

In order to simulate the time dimension of neurons, we also set eight simulation time steps. The category with more discharge times in the set eight simulation time steps was counted as the prediction category.

## 3. Results

### 3.1. Experiment Setup

Our RGC-SNN was trained and tested on burn image dataset. We had set up 1000 epochs, and the training data of each epoch were shuffled. Adaptive Moment Estimation (Adam) [40] with a learning rate of 0.0001 was chosen as the optimizer and cross entropy as the loss function. Our experiments were performed on a computer with NVIDIA GEFORCE RTX-2080Ti GPU with 11 GB of memory. The Pytorch deep learning framework was used to train the network.

### 3.2. Metrics

In this study, we used three main metrics to evaluate the performance of our model: pixel accuracy (PA), mean intersection-over-union (mean IOU), and dice coefficient (DC). PA was the ratio of pixels of the correct class to all pixels. Mean IOU was the average ratio of the intersection and union of actual and predicted regions for different classes. DC was used to evaluate the similarity between actual labels and network predictions. Three indicators were described as Equation (7):

(7) $$\begin{array}{c} PA=\sum_{i=1}^{n}\frac{p_{ii}}{p_{i}}\\ Mean\;IOU=\frac{1}{n}\sum_{i=1}^{n}\frac{p_{ii}}{\sum_{j=1}^{n}p_{ij}+\sum_{j=1}^{n}p_{ji}-p_{ii}}\\ DC=\frac{1}{n}\sum_{i=1}^{n}\frac{2P_{ii}}{P_{ij}+2P_{ii}+P_{ji}} \end{array}$$

where n was the number of all classes; $p_{i}$ was the total number of pixels of class i (i∈{0,1}); $p_{ii}$ was the number of pixels of class i classified as class i; $p_{ij}$ and $p_{ji}$ were interpreted as false positives and false negatives, indicating that the class i sample predicted by the model is class j and the j sample predicted by the model is i, respectively. 

### 3.3. RGC Model vs. LIF Model

We first evaluated the performance of RGC model and LIF model. In order to compare them fairly, we have chosen to test the two models on MNIST [41] dataset, N-MNIST [42] dataset, and DVS-128 gesture [43] dataset. The MNIST dataset contains ten different classes, the handwritten digits 0–9, of which 60,000 were training dataset and 10,000 testing dataset. The N-MNIST dataset imitates biological saccades for recording the complete MNIST dataset with a DVS sensor. DVS-128 gesture dataset is an event-based human gesture dataset. A set of 11 hand and arm gestures was recorded from 29 subjects with 122 trails under three various lighting conditions. N-MNIST dataset and DVS-128 geometry dataset are two typical neuromorphic datasets.

We used the same network structure with state-of-the-art performance at present [39]. Table 1 provides the network structures for each dataset.

**Figure 13:**
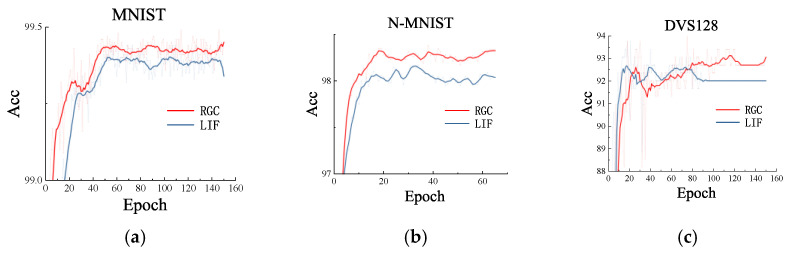
Comparison of accuracy curve during training between RGC model and LIF model. From left to right (**a**) MNIST dataset. (**b**) N-MNIST dataset. (**c**) DVS128 gesture dataset.

In Figure 13, we showed performance curve of the accuracy of RGC-SNN (red line) and LIF Model (blue line) respectively on each testing dataset. It was obvious that the recognition ability of SNN based on RGC model was higher than that based on LIF model. In addition, we also found that our SNN based on RGC model had faster convergence ability. In the classification task, RGC model with more real neuron dynamics maybe better than LIF model.

### 3.4. Segmentation Results

**Figure 14:**
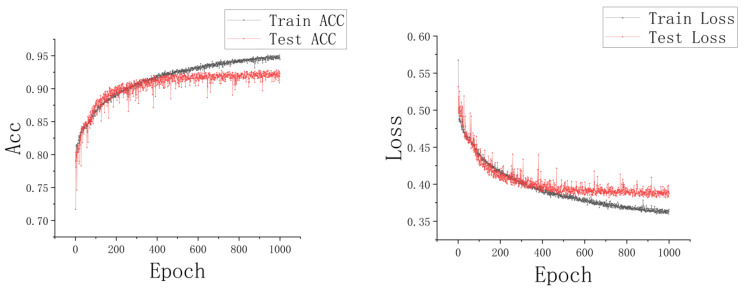
The loss and accuracy changing condition during the training process.

In our segmentation task, our RGC-SNN model (shown in Figure 6) was trained and tested in our burn image dataset. Figure 14 shows the changes of accuracy and loss function as the number of training iterations increases.

The results showed that pixel accuracy increases, the loss gradually decreases with the epoch. After 100 epochs, the corresponding curve tends to be flat. It could be seen that the training curve and test curve in Figure 14 were close to each other. Moreover, we can find that the accuracy of the training dataset was slightly lower than that of the testing dataset at the early stage of training. After our analysis, we found that this phenomenon was due to the fact that the model was in the non-fitting stage in the early training process. Because the training dataset has undergone a series of data augmentation, such as translation, color jitter, and noise addition, and the complex augmentation operations lead to the unstable distribution of the characteristics of the training dataset, so the accuracy of the training set is lower than the testing dataset. Finally, Figure 14 indicates that our model shows no overfitting in the training process, and the model was convergent. The best accuracy of our model in the testing dataset was 92.89%.

**Figure 15:**
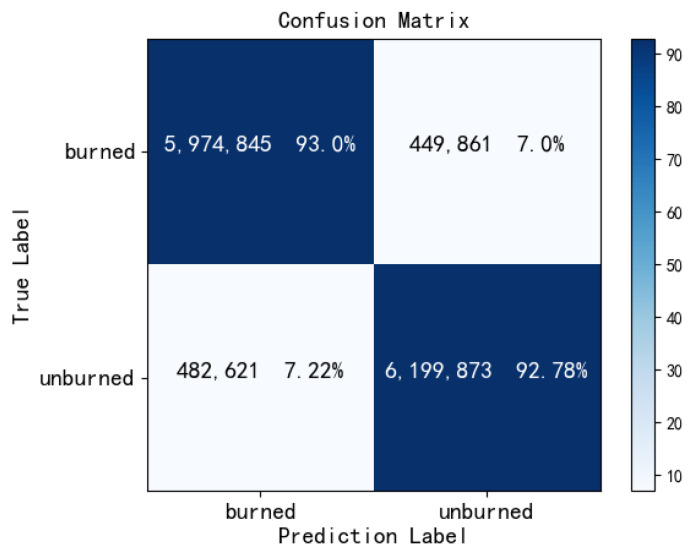
Confusion matrix on test dataset.

At the same time, we calculated the evaluation metrics on the testing dataset. In the segmentation for the burn and unburn areas, our model respectively achieved the results of 0.9289, 0.8792, and 0.8981 on the PA, IOU, and DC metrics. To directly illustrate the performance of RGC-SNN in burn region segmentation, we also calculated the confusion matrix as shown in Figure 15. Confusion matrix intuitively shows whether our model prediction had sample prediction bias. Obviously, our model prediction was relatively balanced, the values of the sub-diagonals are close to each other.

**Figure 16:**
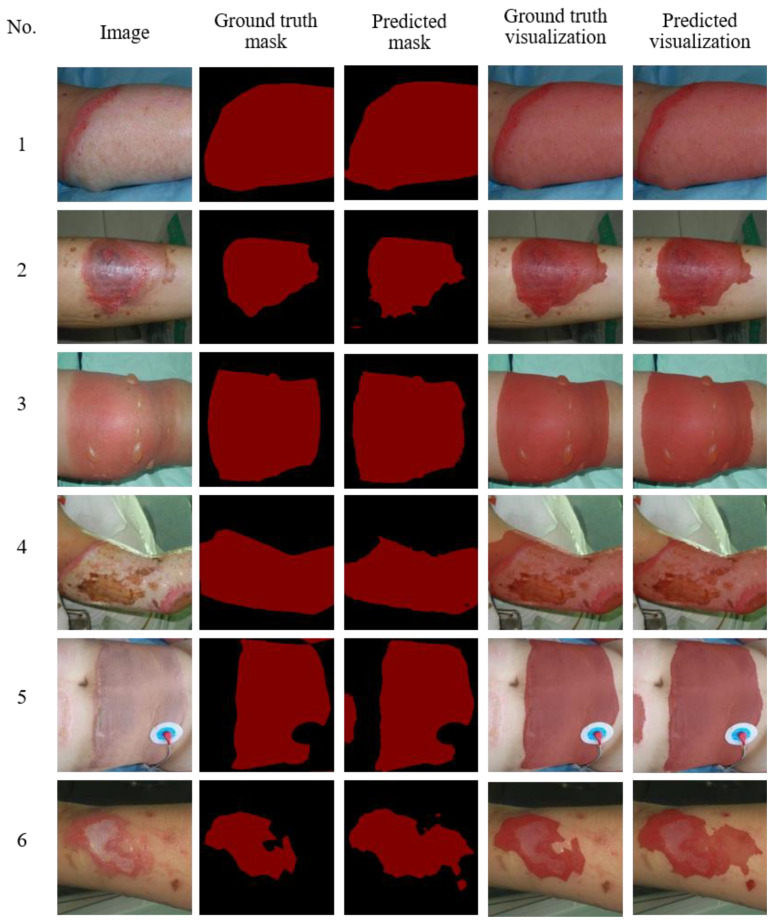
Examples of visualized the prediction results predicted by the RGC network. Red indicates burn areas, and black indicates no burn areas.

In order to show the performance of our model more intuitively, as shown in Figure 16, we used a visual way to show the comparison between ground truth and predicted. Among them, the first column displays original input image, the second column displays real label, the third column displays the prediction result, the fourth column displays ground truth mask overlay with original input image, and the fifth column displays predicted mask overlay with original input image. First of all, RGC-SNN had a greatly segmentation performance on the burn center area. Second, our RGC-SNN performs better than expert annotation in segmenting the edges of burn area. The boundary between the burn area and normal skin in complex burns was often unclear and primarily irregular. In this case, burn specialists prefer to mark the burn area with a straight or circular line. Then, the network tends to segment edge regions more finely (2, 3 and 4 in Figure 16). Finally, we also observed that burn experts often only pay attention to the main large burn areas in burn images, when annotating data, ignoring some minor or marginal burn areas. However, our RGC-SNN may also notice small burn areas outside of large burn areas (5 and 6 in Figure 16).

**Figure 17:**
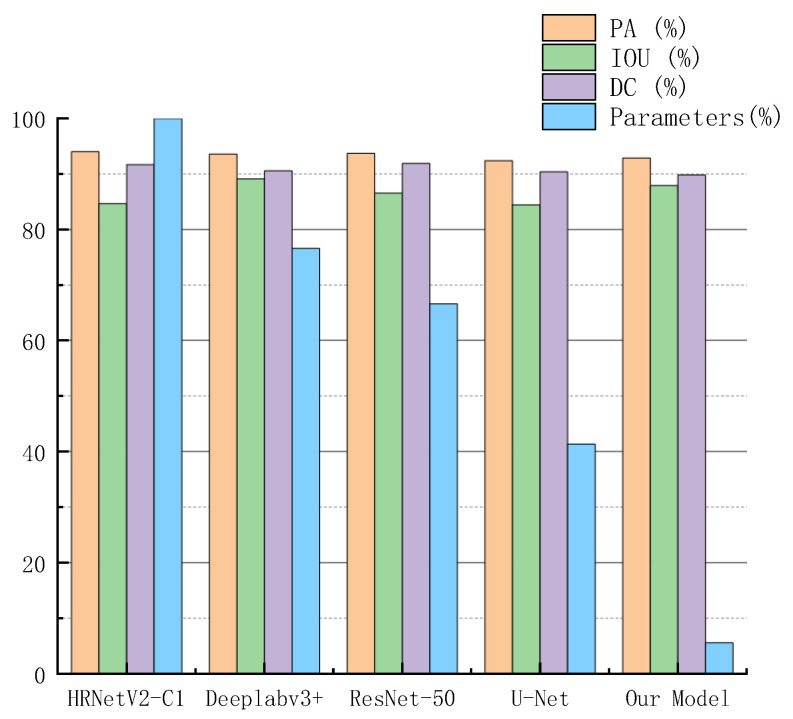
Metrics results of different networks on testing dataset. Among them, we use the largest model parameter quantity as the base number to calculate the percentage of parameter quantity.

In recent years, Deeplabv3+ [44], U-Net [36], HRNetV2-C1 Aug [18], and so on have achieved good performances in many segmentation tasks. In order to intuitively compare the performance of RGC-SNN, we summarized the performance of our model and above methods on our burn image dataset, as shown in Figure 17.

Our RGC-SNN model parameters quantity was only 16.6 Mbytes, on the premise that the parameter quantity was reduced by 73.39% to 94.40%, the accuracy is only lost by 1.14%. Edge computing devices usually had limited memory space and computing resources. Obviously, our model greatly reduced the size of the model on the basis of ensuring that it does not lose the high recognition accuracy of mainstream networks, which was very meaningful for the terminal algorithm.

## 4. Discussion

Clinically, TBSA% of burn wound is an important index to evaluate the amount of resuscitation fluid required by burn patients. Accurate segmentation of burn wounds can quantitatively evaluate TBSA% and help doctors design appropriate rehydration schemes. In this paper, we proposed a SNN based on RGC model to segment burn wound area. RGC-SNN adopted U-type structure to enhance feature reuse capability. The SC module was introduced to suppress the gradient disappearance problem in SNN.

In order to evaluate the performance of the proposed RGC-SNN, a series of comparative experiments were carried out to prove the performance of the proposed model. At the same time, the parameters size of the model was calculated. Visualization results and quantitative evaluation results show that RGC-SNN had the potential to segment burn areas, especially at the edge of burn areas.

Although the segmentation results were excellent, the proposed model still has some limitations. First of all, it was difficult for even burn experts and doctors to accurately outline the image of the burn area. Therefore, this data annotation problem will have a negative impact on network performance. In the future, we will consider using semi-supervised learning strategies to improve the marking and training process of burn image datasets, so as to reduce the dependence on accurate annotation of contours. Second, we tried to quantify the proposed model and further reduced the number of parameters by sacrificing a certain segmentation accuracy. However, the result was not ideal. In the future, we will consider adopting a quantization or pruning method more suitable for SNN to improve the hardware friendliness of our model. In addition, we will collect more burn image data and combine our model algorithm to further improve the performance of burn region segmentation.

## 5. Conclusions

This paper presented a SNN based on RGC model for burn image segmentation. In the segmentation of burned and unburned areas, the network has good performance, reaching the best results of 0.9289, 0.8792, and 0.8981 in PA, IOU, and DC indexes respectively. Moreover, our network parameter quantity was only 16.6M, which was 5.60–13.54% of the mainstream network parameters quantity. Through our RGC-SNN burn area segmentation model, we segment the burn area through the burn patient image to calculate the TBSA percentage of burn patients, which had high clinical value. Our RGC-SNN has better hardware affinity than the mainstream CNNs model, which made our model easy to carry on the edge hardware. Moreover, it provided important help for the development of portable, lightweight and low-cost TBSA percentage computing devices. Our method had been widely recognized and played an important role in the whole burn diagnosis workflow. At the same time, our model had great application value for the terminal of image segmentation in the future.

In the future work, considering that the current model needs more epoch to converge in the training process, we will consider proposing a method to accelerate the convergence. Second, we will also consider using neural network quantification or neural architecture search and other methods to further make our model more lightweight. Similarly, we will also develop edge handheld mobile terminals, and carry our burn area segmentation model on the terminal to assist in burn area diagnosis.

## Figures and Tables

**Table 1:** Network structures used for different dataset.

Dataset	Network Structures
MNIST	Encoding-RGC(LIF)-AP-RGC(LIF)-AP-RGC(LIF)-RGC(LIF)-Out
N-MNIST	Encoding-RGC(LIF)-RGC(LIF)-Out
DVS-128 gesture	Encoding-AP-RGC(LIF)-RGC(LIF)-AP-RGC(LIF)-AP-RGC(LIF)-Out
